# EMG Dataset for Gesture Recognition with Arm Translation

**DOI:** 10.1038/s41597-024-04296-8

**Published:** 2025-01-17

**Authors:** Iris Kyranou, Katarzyna Szymaniak, Kianoush Nazarpour

**Affiliations:** School of Informatics, The University of Edinburgh, Edinburgh, EH8 9AB United Kingdom

**Keywords:** Biomedical engineering, Electrical and electronic engineering

## Abstract

Myoelectric control has emerged as a promising approach for a wide range of applications, including controlling limb prosthetics, teleoperating robots and enabling immersive interactions in the Metaverse. However, the accuracy and robustness of myoelectric control systems are often affected by various factors, including muscle fatigue, perspiration, drifts in electrode positions and changes in arm position. The latter has received less attention despite its significant impact on signal quality and decoding accuracy. To address this gap, we present a novel dataset of surface electromyographic (EMG) signals captured from multiple arm positions. This dataset, comprising EMG and hand kinematics data from 8 participants performing 6 different hand gestures, provides a comprehensive resource for investigating position-invariant myoelectric control decoding algorithms. We envision this dataset to serve as a valuable resource for both training and benchmark arm position-invariant myoelectric control algorithms. Additionally, to expand the publicly available data capturing the variability of EMG signals across diverse arm positions, we propose a novel data acquisition protocol that can be utilized for future data collection.

## Background & Summary

Surface electromyographic (EMG) signals are widely used in academic research on hand gesture recognition for upper limb prosthetic control^1–6^, rehabilitation exoskeletons^7–10^, robotic teleoperation^11–15^ and more recently incorporated into extended reality (XR) applications^16–19^.

Research in upper-limb prosthetics has traditionally used the EMG signals of the participant’s forearm muscles, while they simulate performing different grasps. These signals are then used to train a grasp classifier, which in turn identifies grasp intentions and drives the prosthesis. Similarly, EMG signals are used in various teleoperation applications. Although academic research has resulted in the successful classification of up to 52 grasp classes^20^ in offline settings, clinical solutions give only limited capabilities to the user and this involves frequent re-calibrations, as can be seen in the solutions offered by Coapt, LLC. This mismatch is induced by various factors that in controlled settings are minimised.

More recently, gesture recognition for human-computer interactions (HCI) has served as a source of inspiration for extended reality applications and the metaverse^21^. The use of EMG-based grasp classification in immersive interfaces that use Virtual/Augmented Reality (VR/AR) has seen an increase in popularity in recent years^19,22–31^. As research interest in this area grows, Meta in 2021 revealed their vision of the metaverse and introduced their EMG-powered wrist-based platform^32^. Designing multimodal HCI systems (vision and sensor data combined) is seen as synergistic, leading to investigating sensor data robustness, such as glove, inertial measurement units (IMU), EMG, and other haptic technologies.

Sensor-based solutions can provide a rich source of information. However, signal quality and pattern variability can be influenced by many intrinsic and extrinsic factors. Specifically in the case of EMG signals, these factors include muscle fatigue, perspiration, changes in electrode positions, changes in the position of the participant’s arm, or a combination thereof^33–36^. Several studies focused on how to mitigate the effect of such changes in inter-subject settings when the signal is recorded over a long period of time, between different sessions on the same day, between different days, or between different arm positions in space^37–39^. Specifically, the effect of posture change and arm position has been repeatedly shown to reduce the robustness of the myoelectric control system, which, in effect, hinders the adaptation of such systems for real-life applications^40–52^.

To limit the effect of arm translation, previous research has focused on two main factors. First, participant training combined with feedback has been shown to minimise the signal variability caused by limb position^52^. Second, from a computational perspective, the most popular approach is to record data that capture the aforementioned variability, by recording data from various positions and utilising machine learning techniques that compensate for the effect of arm position^38,40,41,46,53^. Those two approaches are not mutually exclusive and both benefit from a large database of EMG signals during which the arm position factor has been considered. Such a dataset will lead to the development of more advanced and more robust machine learning paradigms, which in turn would lead biofeedback paradigms faster and possibly more intuitive.

The first step in creating robust algorithms in the presence of signal drifts is to create a dataset that captures such shifts. Several datasets from a range of recording conditions have been compiled and published. Table 1 summarizes the available online data sets that contain EMG recordings of multiple participants, performing a set of predetermined grasps under the causes of signal variation mentioned above. For the scope of this paper, we focus only on the datasets that are recorded in any of the three following cases: A) two or more sessions in a day, B) a period of more than one day, and C) various spatial arm orientations.

**Table 1:** Table summarising the existing online datasets that contain recordings from different positions, from different sessions in one day or over the period of multiple days.

Dataset	Limb Positions	# Days	# Sessions per day	Trials per session	Gestures	Subjects	EMG Channels
Khushaba *et al*.^40^	5	1	1	6	8	11	7
Hahne *et al*.^53^	5	1	1	25-35	6	10	96
Palermo *et al*.^59^	1	5	2	12	7	10	14
GRABMyo^57^	1	3	1	7	17	43	28
PutEMG^55^	1	2	1	20	8	44	24
Côté-Allard *et al*.^58^	1	3-4	1	4	11	20	10
ISRMYO-I^60^	1	10	2^†^	1	13	6	16
CapgMyo DB-b^56^	1	2	1	10	8	10	128
MeganePro DB1^61^	2^‡^	1	1	8	10	45	12
MyoBit^74^	2	3	1	1	7	24	16
SeNic^54^	3	3/10	1	3	8	36^§^	8
This Dataset^64^	9	2^†^	2^†^	5	6	8	16

^†^ Not removed the sensors between 2 sessions of same day.

^∤^ Only one position is the same between different days.

^‡^ this refers to the recording in seated and standing positions. They also allow the grabbing objests from different locations on the table.

^§^ Only a subset of the participants are used to record different aspects of the dataset.

At the time of writing, there are only three datasets containing EMG data recorded from various limb positions created with the goal of investigating the effect of limb position, also known as arm translation, on the recorded EMG signal. Khushaba *et al*.^40^ and Hahne *et al*.^53^ record EMG signals from five distinct limb positions in a vertical plane. The biggest difference between these two datasets is the number of sensors used. Khushaba *et al*.^40^ utilised seven sensors located around the forearm, while Hahne *et al*.^53^ employed a forearm band consisting of an array of 96 sensors. Finally, the SeNic^54^ dataset includes recordings of 7 grasps from 3 different arm positions: with the elbow bent and supported on a table, unsupported bent elbow and adjacent to the participant’s body. In terms of datasets involving recordings from multiple sessions or spanning across various days, there is a broader selection available online. The duration of these recordings ranges from two to a maximum of ten days^55–60^. Most of these data sets are recorded in a static setting, with the arm adjacent to the body over the entire period of the experiment. In particular, in Côté-Allard *et al*.^58^ the training recording occurs in one position, but the test data include recordings from a range of angles in space. Since the target of the arm position is determined with respect to the Virtual Reality reference point, it gives the participant freedom of movement to reach the target without many constraints. Consequently, it lacks structured information about arm positioning, which makes it difficult to use in future studies for arm translation.

A more dynamic approach is followed for the recording of the MeganePro DB1 data set^61^, where the recordings capture dynamic movements in both vertical and horizontal planes. Due to the object’s location, the experiment does not explore the spatial resolution in a structured way. The primary focus of the MeganePro DB1 is not arm translation analysis, but rather combining eye tracking with EMG for the purposes of object detection and grasp activation.

Our contribution with the recorded data set encompasses several key aspects in capturing the shifts in the EMG signals due to arm translation. Firstly, we designed a 3 × 3 grid that includes a horizontal plane, complementing the data sets and experiments that only include a vertical plane movement^40,53^. We provide a structured and systematic format to facilitate extensive data analysis with precise annotations for arm translation and grasp activation. Furthermore, to minimise bias and optimise our dataset’s utility, we ensure the data is balanced across all the positions. Lastly, we conducted our experiments over a span of two days, allowing more variability of the data and its implications.

## Methods

### Subjects and Ethical Requirements

The data was recorded from the right hand of 8 intact subjects, all men, over a period of two consecutive days. All participants received a written and oral explanation of the experiment protocol and signed an informed consent form. The experiment was conducted according to the Declaration of Helsinki principles for medical research involving human subjects^62^ and it was approved by the Ethics Commission of the University of Edinburgh, ethics approval number: 2019/89177.

### Acquisition apparatus

The data set consists of recordings of hand kinematics and electrical muscle activity, while the subjects perform specific hand gestures. The apparatus used in the study consisted of a motion capture dataglove and a surface EMG recording system connected to a single laptop responsible for data acquisition.

**Fig. 1:**
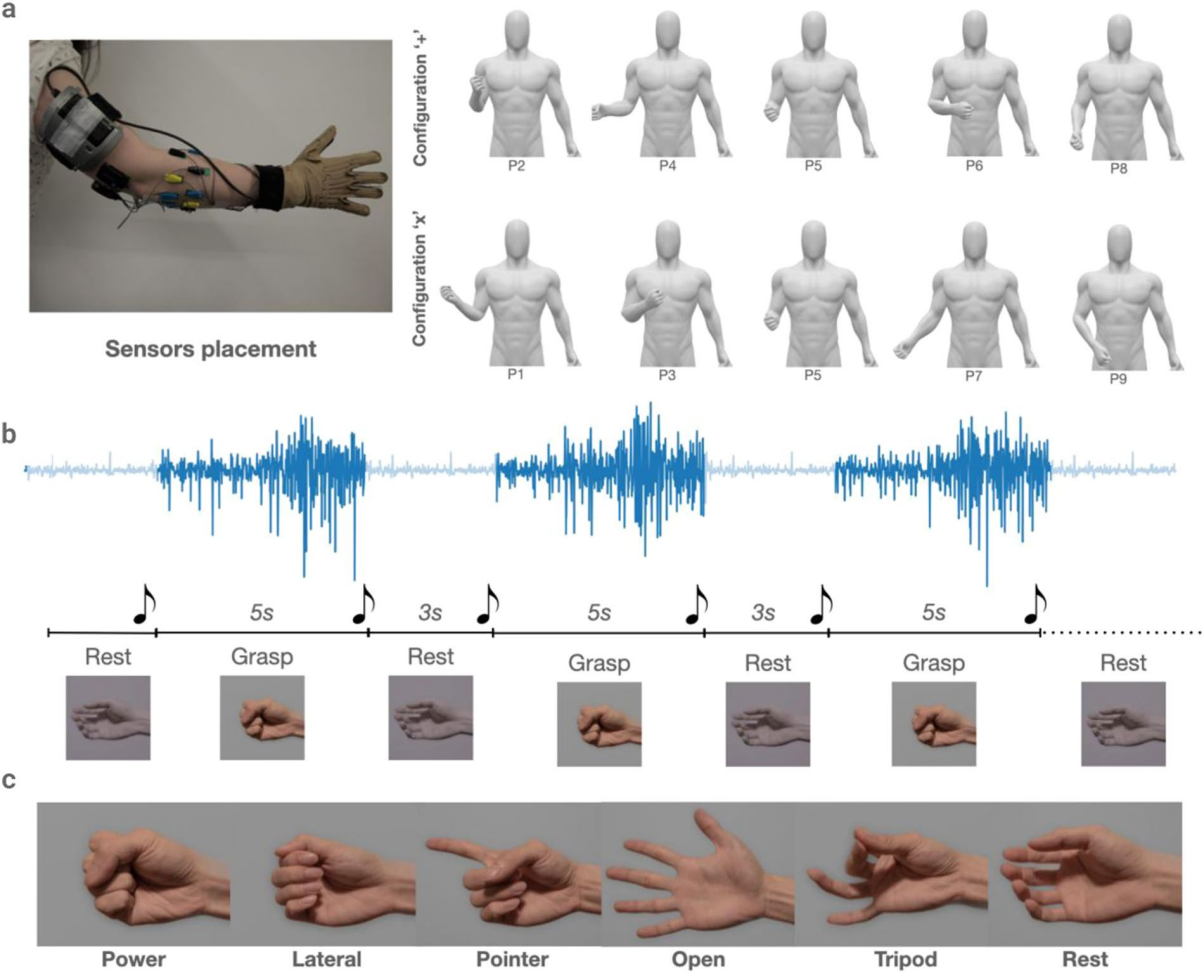
Figure representing the recording protocol. The first row (Part a) shows the sensors positioning on the hand and arm of the participant (we have acquired informed consent to publish the participant’s likeness) and the nine different positions that the participant is asked to move their arm while recording the dataset. Configuration *Con*_+_ is always recorded on day 1 and Configuration *Con*_*x*_ is always recorded on day 2. Part b shows the signal recording process; after a period of rest, during which the signal is not recorded (indicated with light blue color) the participant is prompted to perform a grasp indicated by a visual cue and a beep sound. The participant is required to hold the grasp for 5 sec, followed by 3 sec of rest. This process corresponds to one trial and is repeated 5 times for a predefined grasp and position. The grasps that are recorded during this experiment are shown in part c.

For the **hand kinematics** recordings a wireless motion capture data glove was used. More specifically, the 18-sensor CyberGlove II data glove (CyberGlove Systems LLC), (www.cyberglovesystems.com/cyberglove-ii), which can be seen in Fig. 1a, was used to record data from two bend sensors on each finger, four abduction sensors, plus sensors measuring thumb crossover, palm arch, wrist flexion, and wrist abduction. The data glove is calibrated with respect to the anatomic differences of each participant to ensure accurate recordings. We performed the calibration once at the beginning of the experiment for each participant following a protocol provided by CyberGlove Systems LLC.

The **muscular activity** is recorded by 4 Trigno Quattro Sensors (Delsys, Inc), (delsys.com/trigno-quattro/), each consisting of a reference electrode and four sEMG recording channels, totaling 16 acquisition channels. The sEMG signals are sampled at a rate of 2 kHz and fixed on the forearm using the Delsys adhesive interface. Before electrode placement, the skin of participants is cleaned with 70% isopropyl alcohol. We then placed the acquisition electrodes in two rows of 8. The first electrode was placed on the extensor carpi ulnaris muscle, identified by palpitation, and the rest of the sensors were placed equidistantly around the forearm without targeting specific muscles. For the second band, the electrodes were placed in between two electrodes from the first row. Two of the reference electrodes of the 4 Quattros we used were placed close to the elbow and the other two near the wrist (as shown in Fig. 1a. The reference electrodes on the wrist are hidden by the data glove in this figure). After visually inspecting the signal quality of all EMG channels in the acquisition software, an elastic band was placed around the electrodes to keep them securely attached during the acquisition period.

### Acquisition protocol

Following the sensors positioning and data glove calibration, participants were presented with a computer interface that instructed them to move their arm at a specific position and perform one of the following grasps: power, lateral, pointer, tripod, open and rest (Fig. 1c shows a picture of those grasps). The software responsible for the computer interface and data acquisition was implemented in Python using the Axopy package^63^.

The participants were visually instructed to position their forearm in one of nine different orientations, a 3 × 3 grid appearing on the monitor. Let *P*_*n*_ be the position, where *n* ∈ {1, …, 9} starts from the top left corner with *P*_1_. Consequently, *P*_5_ refers to the central part of the grid. It is a *neutral* position, that corresponds to the forearm being at a 90^°^ angle with respect to the upper arm and parallel to the ground. The rest of the positions were reached by moving the forearm at a 45^°^ angle from this *neutral* position towards the direction indicated by the computer prompt. For example, *P*_2_ required the participant to move their forearm 45^°^ up, and *P*_1_ required 45^°^ upward and 45^°^ left, always with the *neutral* *P*_5_ position as reference. The participants were instructed to keep their upper arm in a stable but relaxed position. The nine different positions are presented in the right half of Fig. 1a. After the participants became familiar with the positions and the six grasps in Fig. 1c the recording started.

Then, the graphical user interface was launched where the image of the desired grasp appeared only on the cell in the 3 × 3 grid that corresponded to the prompted position. In order to eliminate collecting data corresponding to transitioning from the rest phase to grasp, we ensured that prior to each recording the participants’ arm was located in the desired position and the hand formed the prompted grasp correctly. Once the participant was ready, we initiated the data recording.

One ***trial*** in our experiment consisted of 5*sec* holding the prompted grasp, followed by a resting period lasting 3*sec*. This sequence was repeated five times for every grasp in each specific position. Each ***block*** of five trials was then followed by a longer period of rest, during which the participant could relax their arm at any position they chose, for the preferred time duration. The position sequence was predetermined for each day: *Con*_+_: {2,4,5,6,8} and *Con*_*x*_: {1,3,5,7,9} for day 1 and day 2, respectively.

In addition to the visual cue, where the participant was presented with the grasp image in one cell of the grid and the remaining time / repetitions to complete the experiment, an audio cue was used to indicate the start of each 5*sec* recording (sine wave; frequency 400Hz; duration 100ms) and a different audio cue (sine wave; frequency 300Hz; duration 100ms) to indicate the end of the recording and the transition to rest pose. The audio cue to start the recording started 250*ms* before the time our programme started to record, to allow enough reaction time for the participant and avoid recording transitional data from rest to grasp.

The grasp sequence between subjects was pseudorandomised to minimise potential biases. Our data recording protocol shuffles grasps and positions to reduce participants’ fatigue as a result of numerous repetitions of the same movement. Moreover, this makes the experiment less monotonous and boring based on the feedback of the participants.

The first ***session*** of the recordings is completed after 30 trials (5 repetitions of 6 grasps) in all positions, giving us a total of 150 grasp trials. After a longer rest period, the experiment is repeated one more time (second session). The break between the two blocks of recordings was about half an hour long (including the doffing/donning of the dataglove) allowing the subject to leave the room. When the subject returned to the room they were more rested and after some activity (often involving stretching), hence, we report the two blocks recorded in one day as two separate sessions to account for any potential drift that could be present in the signal after the break. The final data set consists of two sessions of five grasp repetitions (trials), in 5 different positions per day (*Con*_+_ or *Con*_*x*_), lasting 2 days.

The between-day recording was not performed with the purpose of capturing the effect of a two-day span experiment, but with the goal of adding variability on the arm positions. However, position 5 (neutral) is one that repeats over the two different days.

## Data Records

The data set is available in the Dryad repository with the name *EMG Dataset for Gesture Recognition with Arm Translation*^64^ and on GitHub (https://github.com/MoveR-Digital-Health-and-Care-Hub/posture_dataset_collection/tree/main/data). This section is the primary source of information on the availability and content of the data described.

The recordings are stored in separate folders per participant, named in the format of ‘participantX_dayY_sessionZ’, where X corresponds to the participant’s unique number ID, *X* ∈ {1, …, 8}, Y to the day of the recording *Y* ∈ {1, 2} and Z to the recorded session of this day *Z* ∈ {1, 2}. Each folder contains the following:

- **Raw EMG Data**: The file named ‘emg_data.hdf5’, contains the raw recordings from the 16 EMG sensors. The file has the format of an HDF5 binary data file and is indexed by the trial number (total of 150 trials). The stored matrix per trial is of 16 x (2kHz*5sec) shape. The signal is processed upon recording, by applying a 4th-order Butterworth filter.
- **Raw Glove Data**: The upsampled, uncalibrated signal recordings of the cyberglove are stored in the file ‘glove_data.hdf5’. The file has a format and size similar to the ‘emg_data.hdf5’ file. It is an HDF5 binary data file with an 18 x (2kHz*5sec) matrix shape per trial.
- **Calibrated Glove Data**: The dataglove correspond to the recordings from each of the 18 sensors. We also calculated the transformation that maps the data to a 5 x (2kHz*5sec) matrix, holding information on the position of each of the five fingers. This information is stored in the ‘finger_data.hdf5’ file.
- **Recording Parameters File**: This is a text file, named ‘recording_parameters.txt’, that includes the configuration details of the recording. It consists of metadata, namely: participant ID (participant_{1-8}), configuration (+ or x), grasp sequence (pseudorandomised sequence of 6 grasps in 5 configuration positions), block and day of recording (1 or 2), and date & time of recording.
  These are followed by a set of parameters used during our experiment, specifically: window size: 150 ms, low cut-off frequency: 20 Hz, high cut-off frequency: 450 Hz, Butterworth filter: 4^*th*^ order, trials number: 5, trial interval time (corresponds to rest time between trials): 3 sec, trial length time (corresponds to the recording time per trial): 5 sec, and the number of channels recorded: 16.
- **Labeling Data**: The raw data set labels are included in the file ‘trials.csv’ and contain the information on the target position and grasp per trial number. Specifically, each entry is a single trial, with trial ID: 1-150, target position: 1-9 (corresponding to grid positions), grasp: 1-6 (corresponding to the grasps), trial number: 0-4, and block number: 0-29 (corresponding to the index of a 5-trial block).

## Technical Validation

### Task Overview

Our objective is to demonstrate the validity and significance of the data set collected. Specifically, we propose a set of experiments which in the future should be referred to as a baseline for arm translation tasks. Overall, our experiments are as follows: 1) Standard classification with data only from a single position, 2) Naive transfer learning classification between positions, 3) Classifying positions for each grasp, 4) Hierarchical multi-label classifier (HMC) with position and grasp dependencies.

### Signal processing and Feature Extraction

The protocol for signal pre-processing was applied to the acquired data to ensure high quality data modelling. During the recording, the data was filtered using a fourth-order Butterworth filter (with low cut-off and high cut-off frequencies of 20 Hz and 450 Hz, respectively). To remove powerline inference, a Notch filter^65^ was applied. After offset correction, we apply a sliding window of 128 ms with a stride of 50 ms. From each window, a set of features and a class label were calculated. To emphasize the universality and feasibility of the data, we choose to extract a well-established set of features commonly used in myoelectric control. Hudgins *et al*.^66^ features constitute mean absolute value, zero crossing, slope sign changes, and waveform length. The grasp activity of a window is assigned a class label based on the majority vote, similar to^67^. Each participant’s data has been processed separately for every session based on the settings we present above. We prepare data by separating it by trials, shuffling, and then splitting it into training and testing data at an 80:20 ratio. Lastly, we normalise data for each sensor using the z-score.

### Classification

In all scenarios we apply a Linear Discriminant Analysis (LDA) classifier which has been shown over the years to be one of the most reliable algorithms used in myoelectric control^1,2,41,68–70^.

#### Standard classification

We start with a naive approach to grasp classification within the same position. This scenario is comparable to other offline pattern recognition-based encoders^40,41,45,46^. For each position, an independent classifier encodes six gestures using 5-fold cross-validation. The results of all subjects are then averaged (the same protocol applies to the remaining scenarios). We achieve on average an accuracy of around 96% in each position. Although these results illustrate an impressive level of classifiability in the conventional approach, we implicitly include certain assumptions, such as data coming from the same distribution. The results are reported in Table 2.

**Table 2:** This table presents a classic scenario of grasp classification with data coming from the same distribution (in our case, the arm position).

*Con*_*x*_	Accuracy	*Con*_+_	Accuracy
Position 1	0.96 *σ*: 0.03	Position 2	0.95 *σ*: 0.04
Position 3	0.96 *σ*: 0.02	Position 4	0.96 *σ*: 0.03
Position 5	0.96 *σ*: 0.02	Position 5	0.96 *σ*: 0.02
Position 7	0.96 *σ*: 0.02	Position 6	0.96 *σ*: 0.02
Position 9	0.96 *σ*: 0.02	Position 8	0.95 *σ*: 0.02

#### Naive transfer learning

The assumption of i.i.d. (independent and identically distributed) in standard machine learning refers to data being independently and randomly sampled from the same underlying distribution. This becomes problematic when the model is presented with new, out-of-distribution data, struggling to encode the unfamiliar patterns in the data. We investigated the effect of arm positioning variance and its significance in translating knowledge between positions. Hence, we conducted an experiment where we trained the model on data from one position and tested on another position from the same configuration (*Con*_+_ or *Con*_*x*_).

The results presented in Table 3 and Table 4 show the accuracy of the independent models trained in the source position *P*_*s*_ (row) and tested in the target position *P*_*t*_ (column) for configurations *Con*_+_ and *Con*_*x*_, respectively. We demonstrate a performance drop of up to 10% in comparison to a standard protocol (training and testing data set from the same position). As expected, in most cases the test on the central position (*P*_5_) results in the highest classification score. In the One-versus-Rest (OVR) setting, the model is trained in a single position (*P*_*S*_) and tested on the remaining positions within this configuration (*P*\*P*_*S*_). In the OVR setting, we also observed that the model train in position *P*_5_ resulted in the highest scores.

**Table 3:** Arm Translation in *Con*_+_: Results for independent models trained on the source position *P*_*s*_ and tested on target position *P*_*t*_ given in each row and column, respectively.

Arm Translation in *Con*_+_						
	Position 2	Position 4	Position 5	Position 6	Position 8	OVR *P*\*P*_*s*_
Position 2		0.88; *σ*: 0.08	**0.91;** σ**: 0.06**	0.87; *σ*: 0.08	0.86; *σ*: 0.08	0.88
Position 4	0.90; *σ*: 0.07		**0.92;** σ**: 0.03**	0.87; *σ*: 0.08	0.88; *σ*: 0.03	0.89
Position 5	0.90; *σ*: 0.06	0.90; *σ*: 0.05		**0.92;** σ**: 0.03**	0.91; *σ*: 0.03	0.91
Position 6	0.89; *σ*: 0.05	0.87; *σ*: 0.04	**0.92;** σ**: 0.03**		0.89; *σ*: 0.03	0.89
Position 8	0.85; *σ*: 0.08	0.87; *σ*: 0.05	**0.90;** σ**: 0.04**	0.88; *σ*: 0.07		0.87

One versus the Rest (OVR) presents a scenario where a model trained on a *P*_*s*_ is tested on remaining positions within this configuration.

**Table 4:** Arm Translation in *Con*_*x*_ results for independent models trained on the source position *P*_*s*_ and tested on target position *P*_*t*_ given in each row and column, respectively.

Arm Translation in *Con*_*x*_						
	Position 1	Position 3	Position 5	Position 7	Position 9	OVR *P*\*P*_*s*_
Position 1		0.88; *σ*: 0.08	**0.91;** ***σ*: 0.05**	0.85; *σ*: 0.07	0.85; *σ*: 0.10	0.87
Position 3	0.90; *σ*: 0.06		**0.92;** ***σ*: 0.03**	0.84; *σ*: 0.07	0.91; *σ*: 0.03	0.89
Position 5	**0.91;** ***σ*: 0.05**	**0.91;** ***σ*: 0.05**		0.89; *σ*: 0.05	**0.91;** ***σ*: 0.05**	0.90
Position 7	0.88; *σ*: 0.06	0.85; *σ*: 0.08	**0.90;** ***σ*: 0.07**		0.88; *σ*: 0.05	0.88
Position 9	0.88; *σ*: 0.05	**0.91;** ***σ*: 0.05**	**0.91;** ***σ*: 0.04**	0.88; *σ*: 0.04		0.90

One versus the Rest (OVR) presents a scenario where a model trained on a *P*_*s*_ is tested on remaining positions within this configuration.

#### Classifying positions for each grasp

We observed a significant performance drop when a model was tested on data from another domain, that is, a different position *P*_*t*_. In transfer learning, two domains are defined as different when we observe either discrepancies in feature space or in marginal distributions. To investigate the cause of the observed behaviour, we took a closer look at arm positioning and its classifiability. By classifying positions (within the given configuration) for each grasp activation, we observed that the classification accuracy was on average around 80%, regardless of gesture. The results are reported in Table 5.

**Table 5:** Position classification in each grasp for both configurations, *Con*_*x*_ and *Con*_+_.

Position classification						
	Power	Lateral	Tripod	Pointer	Open	Rest
Configuration *Con*_*x*_						
Acc.	0.82	0.83	0.81	0.86	0.86	0.79
Configuration *Con*_+_						
Acc.	0.78	0.80	0.73	0.85	0.82	0.73

#### Hierarchical Multi-label Classification (HMC)

**Fig. 2:**
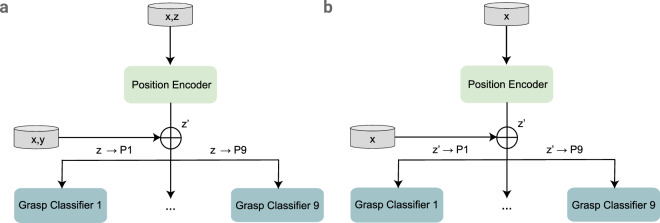
The architecture of the Hierarchical Multi-Label Classification model. In Part a we show the first level in which we train the position encoder *E*_*p*_ with {*x*, *z*} data pairs to predict position ${z}^{{\prime} }$. Here *z* represents the position and *y* the grasp of each sample *x*. We then modify the input vector by concatenating *x* with normalised ${z}^{{\prime} }$ and pass it to train the grasp classifier *E*_*g*_ where *z* value is equivalent to the ground truth in each sample. Part b shows the process in the testing phase. Concretely, once *E*_*p*_ predicts ${z}^{{\prime} }$, the sample $\{x,{z}^{{\prime} }\}$ is then input to the grasp encoder *E*_*g*_, where ${z}^{{\prime} }=g$ (*g* being a grasp). Soft HMC, freezes the constraint ${z}^{{\prime} }\equiv z$ when calculating the accuracy of the model.

Finally, we look at how critical it is to account for differences in arm positioning when trying to identify what type of grasp is being used in everyday situations (such as reaching for a glass of water on a table versus a high shelf). A hierarchical multi-label classifier (HMC)^71^ is applied to leverage position knowledge to classify the gesture. We define the problem as a hierarchically organised tree where each prediction must be coherent with respect to a hierarchy constraint. The hierarchy constraint imposes that a data point assigned to a specific class must also be assigned to all of its predecessors within the hierarchy^71^. Our HMC, shown in Fig. 2, consists of a position encoder (parent node) and leaf nodes ∣*z*∣, corresponding to the nine positions. During the training, data from all positions across both configurations (with position 5 being used only from one configuration) are merged and split in the same manner as in the prior experiments (a.k.a. 80:20 ratio on trials with 5-fold cross-validation). Firstly, we train the position encoder *E*_*p*_ with {*x*_*i*_, *z*_*i*_} data pairs from ${\{{x}_{i},{y}_{i},{z}_{i}\}}_{i=1}^{| D| }$ where *z* represents the position and *y* the grasp of each sample *x* for all dataset samples ∣*D*∣. The augmented features (through concatenation: $\{{x}_{i}\oplus norm({z}_{i}^{{\prime} })\}$) are grouped with respect to the ground truth *z*. Based on *z* corresponding to the nine grasp encoders *E*_*g*:*n*_, nine independent models were trained for grasp classification *y*_*i*_ ∈ {1, . . . , 6}.

During inference time, based on the prediction ${z}^{{\prime} }$ of *E*_*p*_ the sample is assigned to the encoder ${E}_{g:{z}^{{\prime} }}$ to infer grasp activity. We verify the overall performance by calculating the accuracy for every $\{{x}_{i}\oplus norm({z}_{i}^{{\prime} })\}$, using multi-label pairs and their predictions $\{{y}_{i}^{{\prime} },{z}_{i}^{{\prime} }\}$. In the Soft Hierarchical Classification the evaluation metric is modified by freezing the condition on position accuracy, namely ${z}_{i}^{{\prime} }\equiv {z}_{i}$.

Interestingly, we observed a drastic performance drop for HMC compared to standard classification (Table 2), which does not know the position of the arm. Instead of performing grasp classification using train and test data coming from a single position, in the case of HMC, the model is conditioned on the positioning information. Despite data augmentation and incorporation of position information in the data, the multi-label evaluation metric causes a significant drop in performance (Table 6). The position encoder *E*_*p*_ struggles to separate the data in a linear matter as we do not condition on the *y* (grasp), but on the *z* (position), resulting in an accuracy of 58%. Although we can clearly state distribution shifts are present in the data, if we disregard model correctness on arm positioning, the accuracy increases to 92%+ (Table 7). However, we still do not grasp the characteristics and information within the model on arm translation.

**Table 6:** Results from Hierarchical Multi-Label Classifier.

Hierarchical Multi-Label Classification		
*P*_1_: 0.63; *σ*: 0.10	*P*_2_: 0.51; *σ*: 0.07	*P*_3_: 0.63; *σ*: 0.11
*P*_4_: 0.53; *σ*: 0.11	*P*_5_: 0.39; *σ*: 0.07	*P*_6_: 0.53; *σ*: 0.14
*P*_7_: 0.68; *σ*: 0.11	*P*_8_: 0.55; *σ*: 0.12	*P*_9_: 0.61; *σ*: 0.09

In the first level, Position encoder *E*_*p*_, classifies data with an accuracy of 58% and std. 0.05. The table presents classification scores for individual grasp encoders *E*_*g*_ at every position w.r.t. *y* and *z*.

**Table 7:** Results from Soft Hierarchical Classifier.

Soft Hierarchical Classification		
*P*_1_: 0.94; *σ*: 0.3	*P*_2_: 0.92; *σ*: 0.06	*P*_3_: 0.93; *σ*: 0.04
*P*_4_: 0.94; *σ*: 0.02	*P*_5_: 0.94; *σ*: 0.03	*P*_6_: 0.93; *σ*: 0.03
*P*_7_: 0.95; *σ*: 0.02	*P*_8_: 0.94; *σ*: 0.03	*P*_9_: 0.94; *σ*: 0.04

The model follows the same context injection and training protocol as HMC. In this scenario, the Soft Hierarchical Classifier freezes the constraint on position accuracy (${z}^{{\prime} }\equiv z$) and calculates the final score w.r.t. *y*. The accuracy of the *E*_*p*_ is 58%.

## Usage Notes

In this paper, we present the scenario of arm translation in a static setting with a 3 × 3 grid point of reference. To the best of our knowledge, this is the first time a dataset of such position resolution has been presented in the research literature.

We hope that this data set will provide new inspiration and use cases for bridging the gap between data collected in a controlled environment and real-life scenarios. We demonstrated the importance of data variability in arm translation and its impact on the decoding of myoelectric control signals. We envision the following possible applications.

Most of the available online data sets for myoelectric control are collected in a single position. While these established datasets provide reliable information, they lack the representation of variability as a result of arm translation. The effects of arm translation are observed in Tables 3 and 4. Our aim with this data set is to provide a new source of training to address this issue and assist in bridging the gap between myoelectric-based grasp recognition in an experimental setting and a real-life scenario.

We propose a novel design protocol to capture the intricacies of the EMG signal in arm translation. Using a 3 × 3 grid as a reference, we prompt the participant to explore movement in both vertical and horizontal planes.

Current benchmark data sets that include data from different arm positions, such as Khushaba *et al*.^40^ and Hahne *et al*.^53^, have played a critical role in myoelectric control.

We believe that extending the data collection protocol from a single plane to a Cartesian increases the variability of the EMG data. It can serve as a new and challenging benchmark data set for future research to mitigate the adverse effects of arm translation.

Moreover, XR applications are adopting EMG to create a more immersive experience for the user. Such integration of EMG with XR inherits the problems that arise from the EMG instability due to arm translation and variability over different days. Until now, the only publicly available datasets created for XR control that include EMG data are recording gestures from a specific posture. This data set can be used as a benchmark data set for related XR research that attempts to mitigate the disturbances resulting from the variability mentioned above.

The total duration of recording per day of our data set is limited by hardware and human limitations. The myoelectric sensors must be removed and charged after a maximum of three hours of consecutive recording in order to avoid malfunction. This limit was coupled with the participants’ limits of focus and performance before mental and physical fatigue was present.

Existing data sets that record data from a large amount of gestures or more repetitions are most commonly recorded from one arm *supported* position^2,59^. In this study, participants did not rest their hand on the desk. They were asked to keep their arm for a period of approximately 40 seconds in a position that they might not be used to hold their hand in (for example, position 3) while performing the grasp sequence. Our solution to the participant’s fatigue was to allow for enough self-determined breaks and resting phases and break the recording into two sessions, which allowed the participant to move around and relax. This increased the total timing of the experiment, which affects the total mental fatigue of the subject and the draining of the sensors battery. We perceive this to be a potential limitation for all data sets that attempt to record a larger amount of data from a wide range of positions.

An important aspect in the creation of datasets that are used in the field of prosthesis control is the inclusion of data from people with limb difference. The importance of including people with limb differences in myoelectric control experiments has been repeatedly emphasized in recordings of able-bodied people, and people with limb differences typically come from very different distributions^72,73^. The importance of such inclusivity is not limited to prosthesis research and can also benefit XR research.

In addition, our current subject pool consists only of male participants, a practice that may introduce specific biases in the data collection and analysis.

Our data set lacks such recordings, and we perceive this part of our future goals to include information from participants with limb difference and female subjects.

## Data Availability

Examples with data read/write, pre-processing, and a simple classification pipeline are provided in detail in the following repository: https://github.com/MoveR-Digital-Health-and-Care-Hub/MoveR_AT_GREAT. The data collection experiment code can be found on GitHub (https://github.com/MoveR-Digital-Health-and-Care-Hub/posture_dataset_collection) and was implemented in Python using the Axopy package^63^.
